# Regulation of Hepatic Paraoxonase-1 Expression

**DOI:** 10.1155/2012/684010

**Published:** 2012-04-03

**Authors:** Bianca Fuhrman

**Affiliations:** The Lipid Research Laboratory, The Ruth and Bruce Rappaport Faculty of Medicine, Technion—Israel Institute of Technology and Rambam Medical Center, 31096 Haifa, Israel

## Abstract

Serum paraoxonase-1 (PON1) is a member of the paraoxonases family (PON1, PON2, and PON3). PON1 is synthesized and secreted by the liver, and in circulation it is associated with HDL. PON1 has antioxidative properties, which are associated with the enzyme's capability to decrease oxidative stress in atherosclerotic lesions and to attenuate atherosclerosis development. Epidemiological evidence demonstrates that low PON1 activity is associated with increased risk of cardiovascular events and cardiovascular disease and is an independent risk factor for coronary artery disease. Therefore, pharmacological modulation of PON1 activity or PON1 gene expression could constitute a useful approach for preventing atherosclerosis. A primary determinant of serum PON1 levels is the availability of the enzyme for release by the liver, the principal site of PON1 production. Together with the enzyme secretion rate, enzymatic turnover, and protein stability, the level of PON1 gene expression is a major determinant of PON1 status. This paper summarizes recent progress in understanding the regulation of PON1 expression in hepatocytes.

## 1. Introduction

The atherosclerotic lesion is dominated by accumulation of lipid peroxides along with the progression of early plaque development [1]. Serum paraoxonase-1 (PON1) is an HDL-associated lipolactonase, which is synthesized and secreted by the liver [2]. PON1 has antioxidative properties, which are associated with the enzyme's capability to protect LDL [3], as well as HDL [4] from oxidation, to decrease macrophage oxidative status [5], to stimulate cholesterol efflux from macrophages [6], to decrease oxidative status in atherosclerotic lesions [7], and to attenuate atherosclerosis development. Immunohistochemical analysis has revealed accumulation of PON1 in the human atherosclerotic lesion as it progresses from fatty streak to advanced lesion [8]. Recently it was demonstrated that PON1 acts to reduce the oxidizing potency of lipids in atherosclerotic lesions, thus providing protection against oxidation [9]. Epidemiological evidence demonstrates that low PON1 activity is associated with increased risk of cardiovascular events [10] and is an independent risk factor for cardiovascular disease [11].

A variety of nongenetic factors have been shown to influence serum PON1 levels and activity. PON1 undergoes inactivation under oxidative stress and its activity is preserved by dietary antioxidants [12]. Moderate daily consumption of alcohol [13], vitamin C and E [14], wine [15], or pomegranate juice [16], increased serum levels of PON1 in animals and in humans. The level of PON1 in serum is determined mainly by the status of PON1 gene expression in the liver. However, the molecular mechanisms involved in the regulation of hepatic PON1 gene expression were less explored. This paper focuses on nongenetic factors that influence PON1 gene expression in hepatocytes, revealing thus the molecular regulatory mechanisms modulating hepatic PON1 gene expression.

## 2. PON1 Gene Structure

The paraoxonase (PON) gene family consists of three members, PON1, PON2, and PON3, aligned next to each other on chromosome 7 in human and on chromosome 6 in the mouse [17]. In both species the three PONs contain nine exons of approximately the same length. The human PON1 and PON2 genes both have eight introns, and the exon/intron junctions occur at equivalent positions. All PON1s have an extracodon at position 106 (lysine in human PON1). The approximate length of the human PON1 is about 27 kb. In previous studies, Deakin et al. identified a single nucleotide polymorphism (SNP) in the proximal region of the PON1 promoter (C-107T) with an important impact on serum concentrations and activities of the enzyme in different populations [18]. A role for Sp1 and sterol regulatory element-binding protein-2 (SREBP-2) was proposed. A number of potential sterol regulatory element (SRE) sequences exist within the proximal PON1 promoter region that has been shown to be sufficient to respond to statin treatment. The data designate the region around the C-107T polymorphism as being the focus for transcription factor actions and suggest a synergistic effect of Sp1 and SREBP-2 on promoter activity.

## 3. Inflammation

The liver plays a central role in the host response to inflammation, which is associated with a wide array of metabolic changes. These metabolic changes can be induced by the administration of endotoxin (LPS) and by cytokines which mediate the acute phase response, such as TNF and IL-1. LPS and inflammatory cytokines induce composition changes in HDL, due to alterations in hepatic mRNA levels for HDL-related proteins. Administration of LPS and of cytokines in Syrian hamsters resulted in a rapid and marked reduction in PON1 mRNA in the liver, which was sustained as long as 48 hours [19] implicating PON1 as a negative acute phase protein. PON1 was also a negative, acute phase gene in male mice. Within 24 hours of LPS administration, PON-1 mRNA level was reduced by 50% in male mice and increased moderately in female mice, thus showing to be gender dependent. Anti-inflammatory dexamethasone enhanced PON-1 mRNA level by 2-fold in male and female LPS-treated mice and increased PON-1 expression by 8-fold in Hepa cell, a mouse hepatoma cell line [20]. PON1 mRNA expression in hepatocytes was reduced also by oxidized phospholipids found in mildly oxidized LDL through the inflammatory cytokine IL-6, and IL-6 alone produced the same pattern of PON1 mRNA changes [21]. Liver damage induced by CCl_4_ resulted in decreased PON1 gene transcription but increased hepatic PON1 concentration that was related to inhibited protein degradation [22]. Decreased PON1 gene transcription was associated with PPAR*δ* expression. These changes were accompanied by increased hepatic MCP-1 concentration and gene expression, suggesting that PON1 has a hepatoprotective role against inflammation, fibrosis, and liver disease mediated by MCP-1. The interrelationships between PON1 and MCP-1 in the regulation of hepatic inflammation were recently reviewed by Camps et al., [23]. Proinflammatory cytokines, including tumor necrosis factor- (TNF-)*α*, interleukin-1*β*, and interleukin-6 decreased the expression of PON-1 and of apoA-I in hepatocytes by inhibiting PPAR*α* activation [24] and coordinately increased the expression of serum Amyloid A (SAA) via nuclear factor *κ*B (NF-*κ*B) in a manner dependent on both these key transcriptional mediators.

## 4. Oxidation

The atherosclerotic lesion is dominated by accumulation of lipid peroxides along with the progression of early plaque development [25]. Oxidative stress is implicated in atherosclerosis and cardiovascular diseases. Recent lines of evidence appear to support the notion that serum PON1 undergoes inactivation under oxidative stress [12, 26–32]. However, the status of PON1 in the liver and its response to oxidative stress were very poorly investigated. Lipid peroxidation induced by iron-ascorbate decreased PON1 protein in hepatic microsomes derived from humans and rats [33], and this effect was attributable to oxidative stress, because the addition of the BHT antioxidant simultaneously prevented the occurrence of lipid peroxidation and improved the level of PON1 protein.

## 5. Hypolipidemic Drugs

Probucol, a cholesterol-lowering drug with strong antioxidative property, significantly increased serum PON1 concentration and upregulated PON1 mRNA expression in hepatocytes of hypercholesterolemic rabbits [34].

Thus, PON1 gene transcription is modulated by various factors related to inflammation, oxidative stress, or cholesterol. However, the mechanisms of regulation of PON1 gene expression itself remained elusive. Recent studies investigated the molecular mechanisms regulating PON1 gene expression. By comparing the effects of fenofibrate to those statins on PON1 gene expression in hepatocytes, Gouédard et al. characterized the promoter region of the PON1 gene and identified at least one inducer and one class of repressors of the PON1 gene [35]. They have shown that fibrates induced PON1 gene expression and this effect was repressed by PPAR*α* activation, whereas statins inhibited PON1 gene expression via antagonizing the liver X receptor (LXR). On the contrary, Deakin et al. [36] demonstrated that simvastatin upregulated dose-dependently PON1 gene promoter activity, via increasing the nuclear factor sterol regulatory element-binding protein-2 (SREBP-2), which is capable to bind to the PON1 promoter. Clinical studies confirmed these in vitro findings, showing that during statin treatment serum PON1 concentration and activity increased. Complementary studies of the same group revealed that SREBP-2 binds to the PON1 promoter in an interactive manner with Sp1 [36]. Another study presented evidence that Sp1 acts as a positive regulator of PON1 transcription, and that an interaction between Sp1 and protein kinase C (PKC) is a key mechanism for the effect of Sp1 on PON1 gene transcription [37]. The effect of statins on PON1 gene expression was further investigated using a reporter gene assay by measuring luciferase activity of plasmids with a PON1 promoter region transfected into human hepatoma HepG2 cells [38]. Pitavastatin, simvastatin and atorvastatin each significantly increased PON1 promoter activity. Transactivation by pitavastatin was completely abrogated by mithramycin, an inhibitor of Sp1. More recently, the same group of investigators demonstrated that pitavastatin activates the transcription of PON1 gene via phosphorylation of SREBP-2 and stimulation of Sp1 binding to PON1 DNA through the activation of p44/42 MAP kinase signaling cascade [39]. These effects were mediated via PKC activation [40]. Another class of hypolipidemic drugs are the bile acids sequestrates, such as cholestyramine. Based on the findings reported by Gutierrez et al. that bile acids repress PON1 mRNA expression via FXR activation of ileal FGF15 [41], bile acids sequestrates may have beneficial effect on PON1 regulation.

High glucose was also shown to transactivate PON1 promoter through Sp1 activation by PKC in cultured hepatocytes [42].

## 6. Polyphenols

Polyphenols constitute one of the largest category of phytochemicals, most widely distributed among the plant kingdom, and an integral part of the human diet. Dietary consumption of some polyphenols present in wine [43] or in fruit juices increase serum PON1 activity in humans and in mice [44–47]. The mechanisms of action of polyphenols in the upregulation of PON1 were recently investigated by several groups and these studies leaded to elucidation of cellular signal transduction pathways and transcription factors involved in hepatocyte PON1 gene regulation. Quercetin is an ubiquitous flavonoid present in all fruits and vegetables. Dietary quercetin administration to rats was shown to markedly upregulate hepatic PON1 expression at the molecular level [48]. Other dietary polyphenols, such as naringenin, catechin, and quercetin, increased PON1 gene expression by an aryl-hydrocarbon-receptor-(AhR-) dependent mechanism [49]. Resveratrol, a polyphenolic phytoalexin found in grapes and wine, increased PON1 gene expression in human hepatocytes primary cultures and in the HuH7 hepatocytes cell line, and this effect involved a transcriptional mechanism mediated by the unconventional AhR responsive element in the PON1 gene promoter [50]. Berberine, a botanical alkaloid that has been isolated from a number of medicinal plants, has major applications in Chinese medicine. Treatment of HepG2 and HuH7 hepatocytes with berberine increased PON1 expression at the transcriptional level, via a JNK/c-Jun signaling pathway [51]. Pomegranate juice contains polymolecular ellagitannin compounds, such as punicalagins, which are potent antioxidant and antiatherogenic agents [52]. We have recently shown that pomegranate polyphenols mediated stimulation of PON1 gene expression in hepatocytes via cAMP-PKA signaling cascade [53]. Based on previous analysis of the promoter sequence of PON1 gene indicating that it could possibly be regulated by nuclear receptors [35], we have expanded these findings to elucidate a multisteps pathway of the proximal signaling by which pomegranate juice polyphenols can regulate PON1 gene transcription in hepatocytes. Our data show that PPAR*γ* acts as the transcription factor downstream of cAMP-PKA signaling cascade that upregulates PON1 gene transcriptional activity and increases PON1 mRNA expression in hepatocytes [53, 54].

## 7. Urokinase-Type Plasminogen Activator

Direct evidence for PON1 being a target gene of PPAR*γ* evolved from studies investigating atherogenic roles of the urokinase-type plasminogen activator (uPA). uPA is a serine protease enzyme of the fibrinolytic system, and uPA binding to its receptor, uPAR, is implicated in plasmin generation and also in nonproteolytic processes that extend beyond its role in fibrinolysis. We have recently shown that uPA enhanced macrophage atherogenicity by increasing cellular cholesterol accumulation [55] and by promotion of oxidative stress [56]. Very recently we demonstrated that uPA reduces hepatic PON1 gene transcription via its interaction with uPAR on hepatocytes surface. Studies on the mechanism responsible for this effect showed that uPA binding to uPAR stimulates MEK interaction with PPAR*γ* in the nucleus, leading to export of the nuclear PPAR*γ* to the cytosol. By using the chromatin immunoprecipitation (ChIP) assay we have evidenced for the first time that PPAR*γ* binds to DNA sequences in the PON1 promoter region and that uPA reduces the association of PPAR*γ* to PON1 promoter, reducing thus PON1 gene transcription [57].

## 8. Conclusions and Perspectives

There is increasing epidemiological evidence that PON1 protects against development of atherosclerosis. The emphasis in this review was on nongenetic factors that modulate molecular processes related to PON1 gene expression in hepatocytes, reflecting PON1 level and activity in serum. Research in this area has provided mechanistic insight into how PON1 gene transcription can be increased by dietary nutrients. However, as reviewed herein, many of the studies use a single mechanistical approach in a specific model, and thus progression to more pathophysiologically relevant in vitro and animal models is essential. Understanding of these molecular mechanisms is of fundamental importance for atherosclerosis and cardiovascular disorders and could lead to development of novel therapeutical avenues in treatment of these diseases.
